# Land Use and Seasonal Effects on the Soil Microbiome of a Brazilian Dry Forest

**DOI:** 10.3389/fmicb.2019.00648

**Published:** 2019-04-05

**Authors:** Gileno V. Lacerda-Júnior, Melline F. Noronha, Lucélia Cabral, Tiago P. Delforno, Sanderson Tarciso Pereira de Sousa, Paulo I. Fernandes-Júnior, Itamar S. Melo, Valéria M. Oliveira

**Affiliations:** ^1^Brazilian Agricultural Research Corporation, Embrapa Meio Ambiente, Jaguariúna, Brazil; ^2^Division of Microbial Resources (DRM), Chemical, Biological and Agricultural Pluridisciplinary Research Center (CPQBA), Campinas State University (UNICAMP), Campinas, Brazil; ^3^Brazilian Bioethanol Science and Technology Laboratory (CTBE), Brazilian Center for Research in Energy and Materials (CNPEM), Campinas, Brazil; ^4^Brazilian Agricultural Research Corporation, Embrapa Semiárido, Petrolina, Brazil

**Keywords:** Caatinga biome, tropical dry forest, soil microbial communities, metagenomic, seasonality, land use change

## Abstract

Drylands occupy approximately 41% of the Earth’s terrestrial surface. Climate change and land use practices are expected to affect biogeochemical cycling by the soil microbiome in these ecosystems. Understanding how soil microbial community might respond to these drivers is extremely important to mitigate the processes of land degradation and desertification. The Caatinga, an exclusively Brazilian biome composed of an extensive seasonal tropical dry forest, is exposed to variable spatiotemporal rainfall patterns as well as strong human-driven pressures. Herein, an integrated analysis of shotgun metagenomics approach coupled to meteorological data was employed to unravel the impact of seasonality and land use change on soil microbiome from preserved and agriculture-affected experimental fields in Caatinga drylands. Multivariate analysis suggested that microbial communities of preserved soils under seasonal changes were shaped primarily by water deficit, with a strong increase of Actinobacteria and Proteobacteria members in the dry and rainy seasons, respectively. In contrast, nutrient availability notably played a critical role in driving the microbial community in agriculture-affected soils. The strong enrichment of bacterial genera belonging to the poorly-known phylum Acidobacteria (‘*Candidatus* Solibacter’ and ‘*Candidatus* Koribacter’) in soils from dry season affected by ferti-irrigation practices presupposes a contrasting copiotrophic lifestyle and ecological role in mitigating the impact of chemical fertilization. Functional analyses identify overrepresented genes related to osmotic stress response (synthesis of osmoprotectant compounds, accumulation of potassium ions) and preferential carbon and nitrogen utilization when comparing the microbiome of preserved soils under seasonal changes, reflecting differences in the genetic potential for nutrient cycling and C acquisition in the environment. However, the prevalence of nitrosative stress and denitrification functions in irrigation/fertilization-affected soils of the dry season clearly suggest that nutrient input and disruption of natural water regime may impact biogeochemical cycles linked to the microbial processes, with potential impacts on the ecosystem functionality. These findings help to better understand how natural seasonality and agricultural management differentially affect soil microbial ecology from dry forests, providing support for the development of more sustainable land management in dryland ecosystems.

## Introduction

In terrestrial ecosystems, soil microbes play an important role in maintaining structure, decomposing organic matter and nutrient cycling, sequestering of carbon (C) and moderating of climate (Bardgett and Van Der Putten, 2014). With increasing global desertification due to climate change and human-driven practices, it is important to understand the mechanisms that enable soil microbiota to cope with external factors, and their effects on key microbial processes of biogeochemical cycles (Drenovsky et al., 2004; Andrew et al., 2012; Shade et al., 2013).

However, the assessment of soil microbial diversity and their functional contributions is a challenge since 1 g of surface soil may harbor billions of microbes encompassing millions of individual species (Hughes et al., 2001). Recently, advances in culture-independent methods and DNA sequencing technologies have provided a deep understanding of the biotic and abiotic parameters affecting soil microbial community composition; overcoming the limitations of culture-based approaches. Particularly in arid and semi-arid environments, the temperature and water availability have been shown exert over-control on soil microbial communities (Stres et al., 2008; Pasternak et al., 2013; Nielsen and Ball, 2015; Armstrong et al., 2016; Zhao et al., 2016). Short-term water status water status is also an essential factor regulating the microbial activity and microbial community composition (evaluated by PLFA markers) in paddy soils varying in pH, soil organic matter, and soil texture (Liao et al., 2018).

The effects of land use changes and management on soil microbial communities have been widely studied in forests and grassland ecosystems, frequently comparing between tilled or fertilized systems (Rasche et al., 2011; Lauber et al., 2013; Navarrete et al., 2013; Rodrigues et al., 2013; Nacke et al., 2014; Thapa et al., 2018). In dryland regions, the crop production is one of the most devastating land use type nourishing one-third of the global population, and may significantly influence soil health and quality (United Nations Environmental Management Group, 2011). The agricultural management inputs, such as fertilizer, herbicide, and irrigation impact soil microbial diversity and the ecosystem functioning (Ding et al., 2013). Due these water-limited ecosystems cover about 41.5% of the Earth’s land surface (Sorensen, 2009) and have undeniable importance in global biogeochemical cycles (Maestre et al., 2016), studies investigating the effects of environmental parameters, as climate, land use type, and management on the soil microbial communities functions have been requested (Tian et al., 2017; Lüneberg et al., 2018; Pajares et al., 2018). In soils from semi-arid region of Mezquital Valley (P/PET 0.32) the irrigation with different water quantity and quality (freshwater, untreated wastewater, and untreated wastewater) differentially impact the entire and potentially active bacterial community. RNA-based analysis revealed gene abundances involved in nitrogen, carbon and phosphorous cycles are severely affected among different land use systems and season.

These studies become extremely important as the most recent climatic projections suggest an increase of 11–23% in the extent of global drylands by the end of this century (Huang et al., 2016). There is a need to understand how land-use changes affect soil microbial ecology in order to predict ecosystem stability for the development of more sustainable land management in dryland ecosystems.

Seasonal tropical dry forests (STDF) are widely distributed (up to 40% of all tropical forest) and the most threatened and least studied of the world’s forested ecosystems (Murphy and Lugo, 1986; Gillespie et al., 2012). “Caatinga,” an exclusively Brazilian biome composed of the largest STDF in South America, is located in the semi-arid northeastern Brazil (covering about 11% of the territory) harboring more than 23 million of people, indicating high human-driven pressure in the natural environment (Beuchle et al., 2015). In this region, the rainfall is low (below 800 mm year^−1^) and its distribution in the time leads to two well-defined seasons, rainy and dry (Salgado et al., 2015). The Caatinga eco-physiognomy presents a heterogeneous mosaic of thorny trees and shrubs with xerophytic survival characteristics highly adapted to water shortage. For the majority of the tree species the leaves fall in the dry season and white tree trunks and shrubs remain in the landscape (Salgado et al., 2015). Despite harboring great biodiversity and a high level of endemic species, this ecosystem is still poorly studied and preserved (Santos et al., 2011; Pacca et al., 2018). Anthropogenic processes (i.e., agriculture, livestock and predatory extractive) as well as climatic changes have caused worrying environmental damage, such as accelerated desertification and impacts on C and N cycles (de Albuquerque et al., 2012; Menezes et al., 2012; Santos et al., 2014).

Increased attention has recently been paid to the cultivation, characterization and biotechnological potential of Caatinga microbes due to their unique biological features that allow them to survive under severe climate conditions (high temperature, high ultraviolet exposure, and water shortage) (Monteiro et al., 2009; Marcon et al., 2010; Castro et al., 2014; Fernandes-Júnior et al., 2015). Metagenomics-based approaches and 16S rRNA amplicon sequencing have sought to assess the great microbial diversity and shed light on the metabolic strategies that enable the Caatinga microbes to survive under harsh conditions (Lançoni et al., 2013; Nessner Kavamura et al., 2013; Pacchioni et al., 2014; Lacerda Júnior et al., 2017; Leite et al., 2017). Although some studies have shown that soil microbial communities are shaped by seasonality, it is still not clear how microbes adapt to the conversion of intact Caatinga dry forest into cultivation fields, in addition to the underlying impact on the ecosystem functioning.

In this study, the area under investigation is located along São Francisco river valley and includes one preserved area of Caatinga dry forest (Caatinga experimental field) and another one consisting of Caatinga fragments (Bebedouro experimental field) surrounded by agricultural lands and consequently affected by water and nutrients input from cropped areas. These areas represent natural environmental models for investigating seasonality and anthropogenic stressors outlining soil microbial communities under semi-arid conditions. In this context, we hypothesized that: (1) environmental changes associated with seasonal cycles are the major factor outlining the taxonomic and functional traits of the microbiome in preserved soils from Caatinga dry forest; and (2) water and nutrient input by land use practices affect the natural composition and functional capability of soil microbiome, with potential impacts on biogeochemical processes and soil functionality. To test these hypotheses, a field survey was performed combining meteorological and soil chemical parameters with deep metagenome sequencing of the Caatinga dryland soils.

## Materials and Methods

### Study Area and Sampling

**FIGURE 1:**
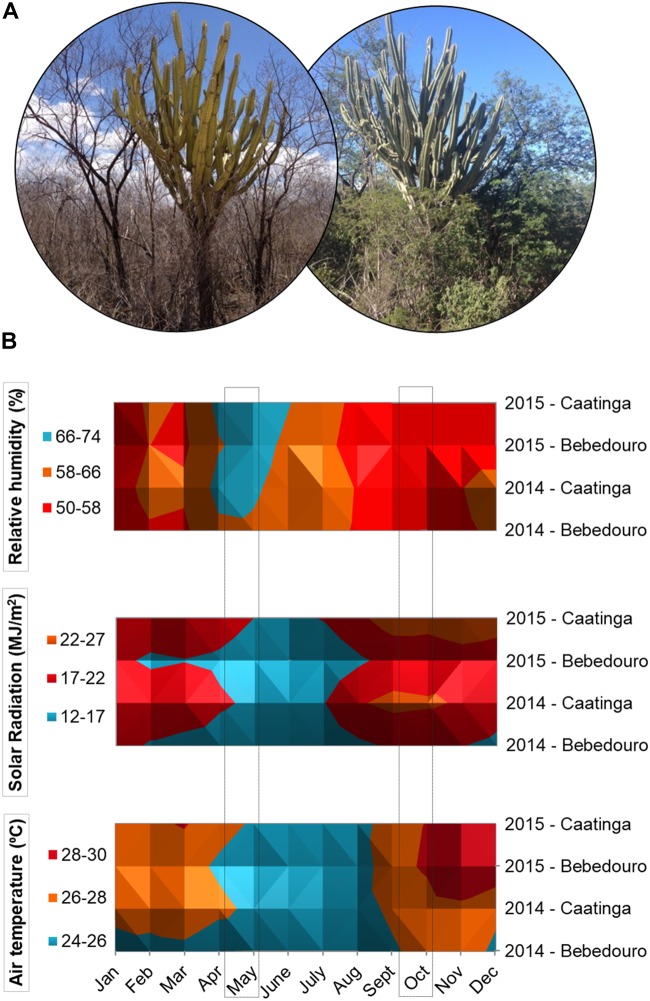
**(A)** Pictures of Caatinga vegetation during dry and rainy season in the semi-arid region of northeastern Brazil (pictures taken by the first author, 2014 and 2015). **(B)** Surface chart showing the distribution of meteorological data of relative humidity (%), air temperature (°C) and solar Radiation (MJ/m2) collected in the CEF and BEF experimental fields during the years 2014 and 2015. Soil samples were collected in October 2014 and May 2015 during dry and rainy seasons, respectively. The data were obtained from automatic meteorological stations of EMBRAPA.

Bulk soil samples were collected at the Caatinga experimental field (CEF) and Bebedouro experimental field (BEF), located in São Francisco River Valley (semi-arid region of northeastern Brazil) at Petrolina city (Pernambuco state, Brazil). Geographic coordinates of the sampling sites and soil characteristics are listed in Supplementary Table S1. CEF is a preserved area of the Caatinga biome managed by Brazilian Agricultural Research Corporation (Embrapa Semiárido, Brazil) widely covered by several dry tolerant species native from Caatinga biome (da Silva et al., 2017; Figure 1A). Within the facilities of Embrapa Semiárido, the CEF had no human interventions at least for the last 43 years, time of the Embrapa settled in the place. BEF encompasses Caatinga fragments surrounded by a management area with irrigation, fertilization and fertirrigation practices during the dry season. The area in BEF is located within an irrigation perimeter with 6000 hectares used for the agricultural crop grown since the 1960s (see Supplementary Figure S1).

Soil samples were randomly collected (depth 0- to 10-cm of the topsoil layer) from three different sites (P1, P2, and P3) across the CEF and BEF areas during the spring (October 2014) and winter (May 2015), which corresponded to the peak of the dry and wet seasons, respectively. Each replicate consisted of five subsamples (arranged 50 m away) which were collected and combined in the field. Samples were stored in a sterile plastic bag, kept refrigerated and transported to the laboratory for storage at −80°C for further DNA extraction. Sampling was authorized by the Institute of Environment and Renewable Natural Resources (IBAMA), process number 02001.004527/2011-90.

### Soil Chemistry and Meteorological Analysis

Chemical measurements were performed in triplicate for each sample. Soil samples were air-dried and sieved through a 2-mm mesh sieve for determination of organic matter (OM), ammonium (N-NH_4_+), nitrate (N-NO_3_-), sulphate (S-SO_4_), potassium (K), calcium (Ca), magnesium (Mg), aluminum (Al), iron (Fe), copper (Cu), boron (B), zinc (Zn), manganese (Mn), sodium (Na) and pH. Chemical analyses were carried out as described by Raij et al. (2001). For soil ammonium (N-NH_4_^+^) and nitrate (N-NO_3_^−^) concentrations, values were determined after extraction of 10 g of soil in 50 mL of KCl (2 M), according to the method described by Keeney and Nelson (1982). In order to obtain a detailed description of the climatic conditions during the samplings, meteorological data were collected daily by automatic stations of Agrometeorology Laboratory (Embrapa Semiárido) in the years 2014 and 2015. Temporal data were collected on air temperature (°C), relative humidity (%), global solar radiation (MJ/m^2^) and rainfall index (mm). Statistical analyses of soil chemical properties were performed by two-way analysis of variance (ANOVA), using the Sisvar 5.0 statistical package (Ferreira, 2011). Statistical differences between means of dry and rainy soils were assessed according to the Bonferroni test (*p*-value < 0.05). All assumptions required by variance analysis were checked.

### Total DNA Extraction and High-Throughput Metagenomic Sequencing

Firstly, soil samples were homogenized by sieving (2 mm) to remove rocks and plant material. Total community DNA was directly extracted using PowerMax Soil DNA Isolation Kit (Mo Bio Laboratories, United States), following manufacturer’s instructions. DNA integrity was checked on 1% agarose gel electrophoresis. DNA quality was measured using NanoDrop spectrophotometer (Thermo Scientific, United States) at A260/280 nm ratio. DNA samples were used to prepare libraries with the Nextera^TM^ DNA Sample Preparation Kit (Illumina^®^-compatible). Environmental DNA samples from each site were barcoded for shotgun sequencing in one single lane of Illumina HiSeq 2000 platform (2 × 100 bp) at Multi-User Laboratory of Functional Genomics (Piracicaba, Brazil), according to the manufacturer’s instructions.

### *In silico* Bioinformatics Analysis

For taxonomic and functional annotation, raw reads of each one of the metagenomes were submitted to the MG-RAST pipeline version 3.6 (Metagenomics Analysis Server Annotation) (Meyer et al., 2008). Paired reads were merged with a minimum overlap of 8 bp and a maximum difference of 10%. Low-quality reads and artificial duplicate reads were removed using MG-RAST quality control with default quality thresholds. The taxonomic assignment of the high-quality unassembled reads was performed via BLASTP search against SEED database (Overbeek et al., 2005) using the lowest common ancestor (LCA)-based algorithm (maximum *e*-value cutoff of 1e^−5^, minimum percentage of identity cutoff of 60% and a minimum alignment length cutoff of 50 base pairs). Functional analysis was performed against subsystems category of SEED database using BLASTP with the same cutoffs.

### Statistical Analysis of Metagenome Data

Statistical analyses were performed using the STAMP (Statistical Analysis of Metagenomic Profiles) software version 2.1.3 (Parks and Beiko, 2010) to identify biologically relevant differences. The input data were relative abundance assigned to the different taxa and functional categories of SEED databases for each metagenome. Statistical differences between soils of the seasonal period (dry versus rainy) from CEF (*n* = 3) and BEF (*n* = 3) were determined by two-sided Fisher’s Exact test, and Storey’s FDR (false discovery rate) method was used for multiple test corrections, as recommended by the STAMP developers. All features with a *p* < 0.05 were filtered. Multivariate analyses of soil chemical variables and taxonomic profiles were performed using the PAST software version 3.15 (Hammer et al., 2001).

## Results and Discussion

### Meteorological Data and Chemical Properties of Soil Samples

**FIGURE 2:**
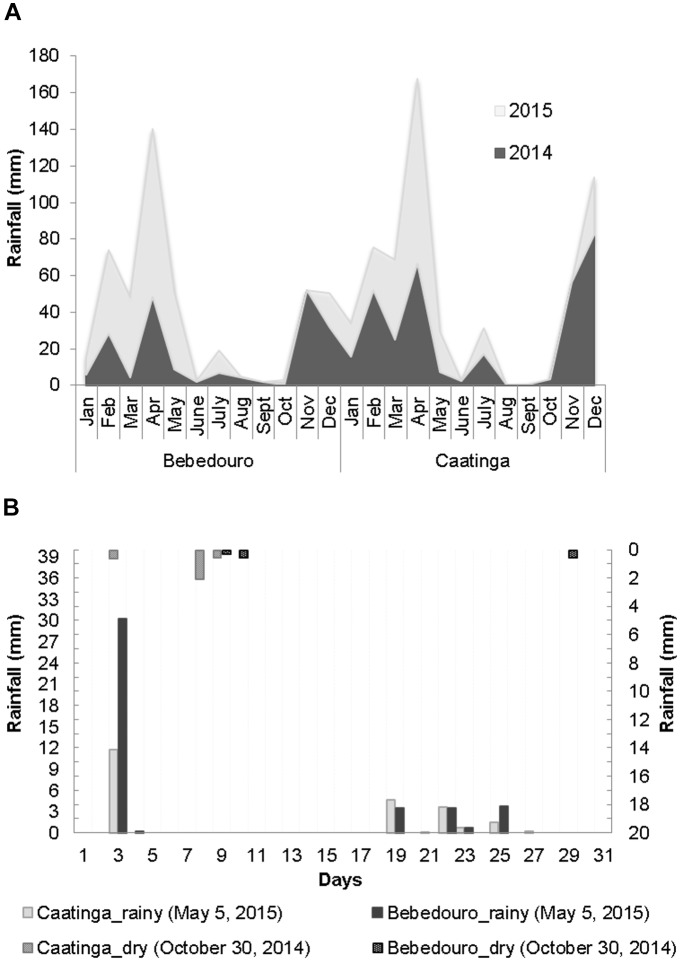
**(A)** Monthly rainfall distribution at the CEF and BEF stations of the Caatinga during the years 2014 and 2015. **(B)** Daily rainfall data (0–30) during soil sampling on 5 May and 30 October, corresponding to the rainy (main axis) and dry (secondary axis) periods, respectively. The data were obtained from automatic meteorological stations of EMBRAP9A.

The climatic patterns of CEF and BEF in the years 2014 and 2015 were relatively consistent with the expected ones for the well-defined dry and rainy seasons of the Caatinga biome (Salgado et al., 2015). Solar radiation and air temperature were higher during August to October 2014 (corresponding to the dry season), while higher air humidity rates were obtained in May 2015 (corresponding to the rainy season) (Figure 1B). With regards to the rainfall, although the intra-seasonal precipitation is dynamics and varies slightly from year to year, the rains were concentrated from December to June (years 2014 and 2015), while a drought period was observed from August to October with very low or even zero rainfall indexes in both experimental fields (Figure 2A). In this work, the daily meteorological monitoring ensured that soil samples were collected after a long drought period (30 October of 2014) and rainfall events (5 May of 2015), during dry and rainy seasons, respectively (Figure 2B).

Chemical characteristics were determined from rainy and dry soils of preserved (CEF) and anthropized (BEF) Caatinga fragments. The soils of BEF, surrounded by crop lands, showed higher content of macronutrients (C, P, N, Ca, S, K, and Mg) and micronutrients (Mn, Cu, Fe, Al, and B) as well as higher values of parameters related to soil fertility such as cation-exchange capacity (CEC), base saturation (V%) and sum of exchangeable bases (SB) when compared to rainy BEF and pristine CEF soils (Table 1).

**Table 1:** Chemical properties of soil samples from Caatinga and Bebedouro experimental fields (*n* = 3).

	Caatinga Experimental Field (CEF)		Bebedouro Experimental Field (BEF)		
Soil parameters	Dry	Rainy	Dry	Rainy	*CV (%)*
pH (CaCl2)	4.9 *aA*^∗^	4.2 *aB*	5.1 *aA*	5.4 *aA*	5.63
P (mg.dm^.3^)	4.0 *aB*	4.0 *aA*	15.0 *aA*	6.0 *bA*	15.33
K (mmolc.dm^.3^)	2.0 *aB*	2.3 *aB*	7.8 *aA*	2.7 *bA*	3.64
Ca (mmolc.dm^.3^)	7.7 *aB*	5.0 *aB*	56.0 *aA*	21.0 *bA*	13.50
Mg (mmolc.dm^.3^)	1.7 *aB*	2.0 *aB*	23.0 *aA*	6.0 *bA*	7.25
H+Al (mmolc.dm^.3^)	20.0 *aB*	21.0 *aA*	34.0 *aA*	13.0 *bB*	11.00
SB (mmolc.dm^.3^)	11.4 *aB*	9.5 *aB*	86.8 *aA*	30.7 *bA*	15.13
CEC (mmolc.dm^.3^)	31.4 *aB*	29.5 *aB*	120.8 *aA*	41.7 *bA*	5.82
V (%)	36.3 *aB*	32.0 *aB*	71.0 *aA*	69.0 *aA*	14.34
B (mg.dm^.3^)	0.2 *aB*	0.2 *aA*	0.7 *aA*	0.3 *bA*	21.33
Cu (mg.dm^.3^)	0.7 *aB*	0.8 *aA*	3.2 *aA*	0.8 *bB*	23.10
Fe (mg.dm^.3^)	17.3 *aB*	29.0 *aA*	114.0 *aA*	25.0 *bA*	32.94
Mn (mg.dm^.3^)	8.2 *aB*	5.2 *aB*	15.5 *aA*	12.2 *aA*	28.63
Zn (mg.dm^.3^)	0.4 *aB*	0.6 *aA*	1.3 *aA*	1.0 *aA*	31.01
OM (g.dm^.3^)	13.7 *aB*	15.0 *aA*	30.0 *aA*	19.0 *aA*	34.61
Total N (mg.kg^.1^)	966 aB	910 aA	1771 aA	1050 bA	12.60
Moisture (%)	0.4 *bB*	3.1 *aB*	2.7 *bA*	4.6 *aA*	26.88

*^∗^Averages followed by the same letters do not differ by Bonferroni t test (p < 0.05) after a two-way ANOVA analysis. The lowercase letter compares the same soil parameter within the same experimental filed, comparing between seasons. Uppercase letters compare the same soil parameter within the same season, comparing the experimental fields. CV = coefficient of variation.*

Water content (%) ratios of 1.67 and 8 were observed between rainy and dry soils from BEF and CEF, respectively (Supplementary Table S2). Likewise, the moisture was the most significantly different (*p* < 0.05) parameter between rainy and dry preserved soils as determined by *F* test (Table 1). Despite the similar rainfall index (Figure 2B), BEF soils collected in the dry season presented remarkably higher and similar moisture as compared to preserved soils of dry (average of 0.4 %) and rainy (average of 3.1 %) seasons, respectively (Table 1). These results suggest that BEF is a good experimental field for the investigation of the impacts of natural water regime alterations and land use practices on the soil microbiome.

### Impacts on the Soil Microbial Community Structure by the Natural Seasonal Regime and Agriculture Practices

**FIGURE 3:**
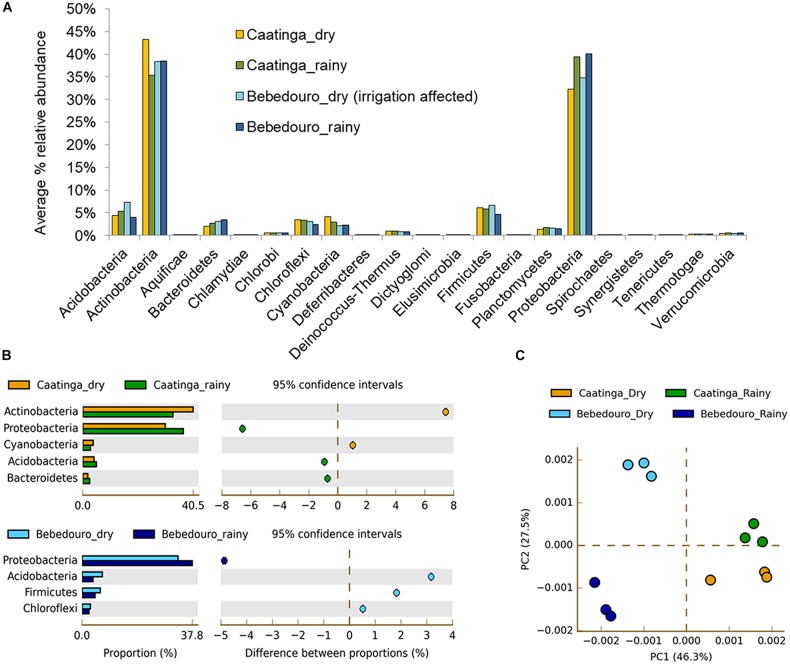
Relative abundances of major bacterial taxonomic groups at the phylum level in Caatinga and Bebedouro experimental fields in dry and rainy seasons **(A)** and **(B)** Microbial phyla that exhibited significant differences (*p* < 0.05) between dry and rainy seasons of CEF and BEF soils. Significance was determined by Fisher’s exact test with Story’s FDR correction for multiple comparisons. Only taxa with difference between proportions > 0.2 (i.e., considered large effect) are shown **(C)** Principal Component Analysis (PCA) based taxonomic profile at genus level.

**FIGURE 4:**
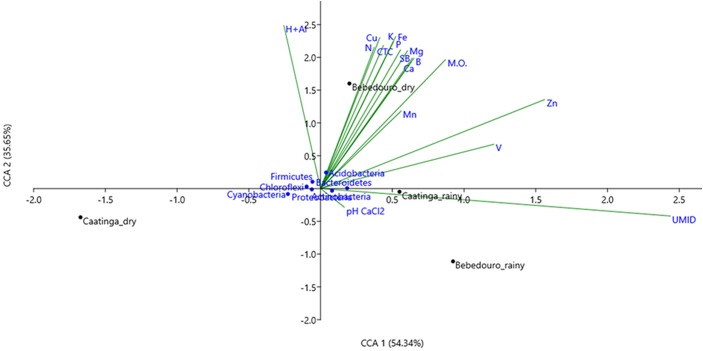
Canonical correspondence analysis (CCA) based on microbial community composition (at phylum level) and soil properties of the soils collected from Caatinga and Bebedouro experimental fields.

It is well known that several biotic (i.e., competitiveness) and abiotic factors can contribute to shaping microbial community structure over time and space (Drenovsky et al., 2004; Andrew et al., 2012; Shade et al., 2013). In arid and semi-arid soils, the temperature and rainfall regime may exert over-control over microbial communities (Stres et al., 2008; Pasternak et al., 2013; Nielsen and Ball, 2015; Armstrong et al., 2016; Zhao et al., 2016). Previous studies based on T-RFLP analysis of 16S rRNA gene have shown that temperature and drought stress caused by seasonal differences were the major forces on the modulation of the microbial community in bulk soil and rhizosphere of *Cereus jamacaru* (cactus) and two leguminous trees (*Mimosa tenuiflora* and *Piptadenia stipulacea*) from the Caatinga biome (Lançoni et al., 2013; Nessner Kavamura et al., 2013). Herein, approximately 400 million (about 40 Gb) high-quality reads were obtained after control quality, with an average length of 100 bp (Supplementary Table S3). Taxonomic classification of reads based on the lowest common ancestor (LCA) algorithm showed that the Bacteria dominated CEF and BEF soil samples in the dry (94.45 and 94.11 %, respectively) and rainy (94.28 and 94.75 %, respectively) seasons. The remaining reads corresponded to Archaea and Eukarya, Viruses and unassigned sequences (Supplementary Figure S2a). Principal component analysis (PCA) based on the taxonomic profiles (at genus level) showed a clear separation between soil microbial communities of preserved (CEF) and agriculture-affected (BEF) sites as well as between the dry and rainy seasons of both experimental fields (Figure 3C). Canonical correspondence analysis (CCA) was used to determine whether correlations in microbial structure were associated with soil chemical parameters. The preserved CEF soils of rainy season tended to cluster with soil humidity; whereas soil micro- and macronutrients content (N, Cu, K, CTC, SB, P, Fe, Mg, Mn, B, Ca, and O.M) contributed most to the variance in BEF soils of the dry season (Figure 4).

These results suggested that community structure in preserve soils is shaped primarily by the natural seasonality factors, whereas nutrient supply seems to be a strongest factor outline the microbial structure in fertilization-affected soils of the dry season.

### Enrichment of Bacterial Groups According to Seasonal Variation and Agriculture Impact

At the phylum level, bacterial composition changed according to the experimental field and seasonal variation (Figure 3A). A significant enrichment (*p* < 0.05) of the phyla Actinobacteria and Cyanobacteria was found in the dry season of CEF soils in contrast to an increase of Proteobacteria, Acidobacteria and Bacteroidetes in the rainy season (Figure 3B). Similar responses of have been described in bacterial communities of soils collected from other sites of Caatinga biome (Lançoni et al., 2013; Nessner Kavamura et al., 2013), as well as in many drylands subjected to rainfall regimes (Barnard et al., 2013; Zhao et al., 2016). Actinobacteria, Proteobacteria and Acidobacteria were also the most responsive phyla in African and Japanese forest soils exposed to drying and rewetting treatments (Zhou et al., 2016). The increase of Actinobacteria in the drought periods is perhaps unsurprising, given their known ability to grow under high temperature, salt concentrations and radiation as well as wide metabolic capacity, which also makes them interesting targets for bioprospecting purposes (Mohammadipanah and Wink, 2016). Similarly, Cyanobacteria members dwelling in arid environments have developed a broad range of strategies for ecological adaptation, including synthesis of extracellular polysaccharides for water retention (Tamaru et al., 2005), protective shield against UV radiation by pigments (Gao and Ye, 2007), and also N_2_-fixation and photosynthesis for nitrogen (Tashyreva and Elster, 2015) and carbon (Harel et al., 2004) supply, respectively. Indeed, the low moisture required to be photosynthetically active supports the increase in the drought period and the hypothesis of a vital role of this photoautotrophic group as primary producers in dryland environments (Harel et al., 2004; Tracy et al., 2010; Lins et al., 2016). On the other hand, Proteobacteria and Bacteroidetes have copiotrophic strategies with quick growth responses to high moisture and resource availability (Fierer et al., 2007).

Interestingly, some similar and differential responses were observed in soils influenced by ferti-irrigation crop area. The phylum Proteobacteria was significantly overrepresented (*p* < 0.05) in BEF soil from the rainy season, while Acidobacteria, Firmicutes and Chloroflexi were more enriched in the dry season (Figure 3B). The non-restrictive water content (Table 1) during the dry season ensured by the irrigation practices may explain the lack of statistical difference (*p* < 0.05) observed in the Cyanobacteria and Actinobacteria abundances among BEF soils. Interesting, Acidobacteria appeared notably more abundant in the dry period of (Figure 3A,B). Given the high nutrient availability in the dry period of BEF soils, the notable enrichment of Acidobacteria disagree with the described oligotrophic life-strategy (analog to K-strategist: slow-growing) of this phylum according to nutrition and growth based-classification (Fierer et al., 2007). However, this scenario is consistent with the different ecological classification proposed more recently by Barnard et al. (2013), where Acidobacteria subgroups displayed a fast life-strategy in response to greater water and nutrient availability in California grasslands soils.

Even with the large proportion of unclassified reads limited by the direct annotation of short reads (Supplementary Table S3), LCA-algorithm was used for phylogenetic resolution at the genus level and provides insights into specific metabolic potential and life-strategy of enriched bacterial groups. Characterized as strictly aerobic, the *Conexibacter* genus (belonging to Actinobacteria Phylum) was the most represented in all soil metagenomes (Supplementary Figure S2b), except in the irrigation-affected soils (BEF) that displayed a greater abundance of ‘*Candidatus* Solibacter’ (Supplementary Figure S2b). Although the ecological role of *Conexibacter* is still not understood, previous metagenomic studies have already reported these microbes as dominant in Caatinga soils (Pacchioni et al., 2014) and quite abundant in other soils (Janssen, 2006; Seki et al., 2012; Deng et al., 2015). Moreover, soil *Conexibacter* strains were reported as slow-growing microorganisms and with high genomic G+C-content (over 70%), key attributes for survival under stressed arid conditions (Mann and Chen, 2010; Seki et al., 2012).

**FIGURE 5:**
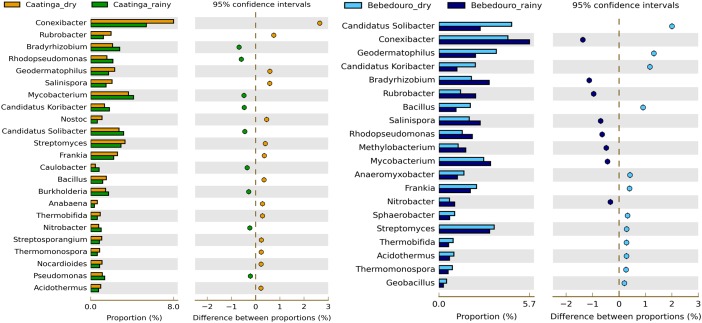
Significant differences in microbial communities at the genus taxonomic levels between dry and rainy seasons of CEF and BEF. Significance was determined by Fisher’s exact test with Story’s FDR correction for multiple comparisons, with corrected *p* < 0.05 significance filter. Only taxa with difference between proportions > 0.2 (i.e., considered large effect) are shown.

Interestingly, with the exception of *Mycobacterium* (enriched in the rainy season), all genera belonging to the phylum Actinobacteria (*Conexibacter, Rubrobacter, Streptomyces, Frankia, Thermobifida, Thermomonospora, Nocardioides* and *Acidothermus*) were highly enriched in the drought period of preserved CEF soils (Figure 5). Most of them are described as polyextremotolerant bacteria remarkably adapted to thrive under harsh conditions (Shivlata and Satyanarayana, 2015). These findings corroborate with previous reports on Actinobacteria members variation in response to irrigation influence, suggesting that this bacterial group may be a good marker for “health” of arid soils (Frenk et al., 2014; Wafula et al., 2015). On the other hand, taxa affiliated to Proteobacteria (*Bradyrhizobium, Rhodopseudomonas, Burkholderia, Nitrobacter* and *Pseudomonas*) and Acidobacteria (‘*Candidatus* Koribacter’ and ‘*Candidatus* Solibacter’) were slightly overrepresented to the rainy season (Figure 5).

Unlike, BEF soils of the dry season impacted by ferti-irrigation practices showed strong enrichment (*p* < 0.05) of ‘*Candidatus* Koribacter’ and ‘*Candidatus* Solibacter’ (belonging to the Acidobacteria phylum) (Figure 5). Although widely abundant and diverse across soils (Janssen, 2006; Fierer et al., 2007; Navarrete et al., 2013). the physiology and ecological roles of the Acidobacteria phylum are poorly understood due to the difficulty to culture representative isolates (Kielak et al., 2016). Despite they were described as oligotrophic and K-strategist lifestyle (Fierer et al., 2007), integrated data derived from physiological, genomic and metagenomic studies gathered from Kielak et al. (2016) have pointed out that different Acidobacteria subdivisions may thrive in non-oligotrophic niches. Likewise, a positive correlation of *Acidobacteria* subgroups and carbon availability was showed in agricultural soils from the Amazon rainforest (Navarrete et al., 2013) and ultramafic soils from a tropical savanna (Pessoa-Filho et al., 2015). Wafula et al. (2015) also reported a positive correlation of total N, organic matter and moisture with *Acidobacteria* communities from treated wastewater-irrigated soils. In fact, bacterial isolates of this phylum are able to grow in high concentrations of C sources (Eichorst et al., 2007; Castro et al., 2014; García-Fraile et al., 2016), and genetic evidence have revealed the presence of genes required to decompose complex substrates (García-Fraile et al., 2016; Kielak et al., 2016; Lacerda Júnior et al., 2017). Genomic insights also presume a role of ‘*Candidatus* Koribacter’ and ‘*Candidatus* Solibacter’ in N_2_ cycling by the reduction of nitrate, nitrite, and possibly nitric oxide (Ward et al., 2009).

All these findings support and shed new insights on the role of the Acidobacteria in the nutrient and carbon cycling in agriculture-impacted soils with high N and organic matter content.

On the other hand, soil community of rainy season was most enriched by *Conexibacter*, *Rubrobacter*, *Salinispora* and *Mycobacterium* (Actinobacteria Phylum), and other genera belonging to Proteobacteria (*Bradyrhizobium*, *Rhodopseudomonas*, *Methylobacterium* and *Nitrobacter*) (Figure 5). It is interesting to note that most of the Proteobacteria-affiliated taxa (especially Alphaproteobacteria) enriched in rainy soils from both experimental fields have generally been characterized as fast-growing and free-living N-fixing and nitrifying bacteria in soil ecosystems (Tsoy et al., 2016). The abundance of *Bradyrhizobium* in both sampling seasons and soils was remarkable. This genus is widely spread in the soils worldwide (Delgado-Baquerizo et al., 2018), including the bulk soil in Brazilian semi-arid lands (Pacchioni et al., 2014). *Bradyrhizobium* also nodulates several crops and native legumes of Brazilian semi-arid (Martins et al., 2003; Leite et al., 2017; Marinho et al., 2017; Menezes et al., 2017; Santos et al., 2017; Rodrigues et al., 2018). The abundance of *Bradyrhizobium* in the soil evaluated, even in low water availability, indicates their drought tolerance and biotechnological potential for rhizobial prospection studies.

*Anaeromyxobacter*, a facultative anaerobic myxobacterium, was the only Proteobacteria-affiliated genus significantly overrepresented (*p* < 0.05, Figure 5) in the soils with land-use influence. *Anaeromyxobacter* spp. have been proposed to contribute to *in situ* bioremediation applications for ferric-iron and uranium reduction, herbicides degradation, and are important in N and C cycling in aquatic, sedimentary, and agricultural soils (He and Sanford, 2003; North et al., 2004; Thomas et al., 2009; Sanford et al., 2012).

The increase of *Anaeromyxobacter spp.* and Acidobacteria groups in agriculture-affected soils may be explained by the process called “priming effect,” generated by the nutrient input. Furthermore, their previously recognized metabolic and genetic potential presume a role in the nutrient cycling (mainly N), mitigating the impacted of the fertilization, such as soil acidification, nitrate leaching, and loss of biodiversity (Ward et al., 2009; Sanford et al., 2012).

### Differences in the Functional Traits of the Microbiome From Preserved Soils Under the Seasonal Regime

The functional composition of Caatinga soil metagenomes was analyzed using high-quality reads obtained after quality control filtering of MG-RAST pipeline. Only 20–25% of total sequences could be assigned to functional categories using the SEED database (Supplementary Table S3). Most of them were assigned to one of the following level 1 SEED subsystems related to housekeeping functions: “carbohydrates,” “protein metabolism,” “amino acids and derivatives” and “RNA and DNA metabolism.” The relatively minor categories represented specific ecological functions such as nutrient cycling, stress response and dormancy/sporulation strategies (Supplementary Figure S3). STAMP software was used in the statistical comparisons at a finer functional classification (SEED level 2 and 3) in order to the understanding of how the functional capabilities encoded by the soil microbial communities are affected in response to seasonal and anthropogenic factors.

**FIGURE 6:**
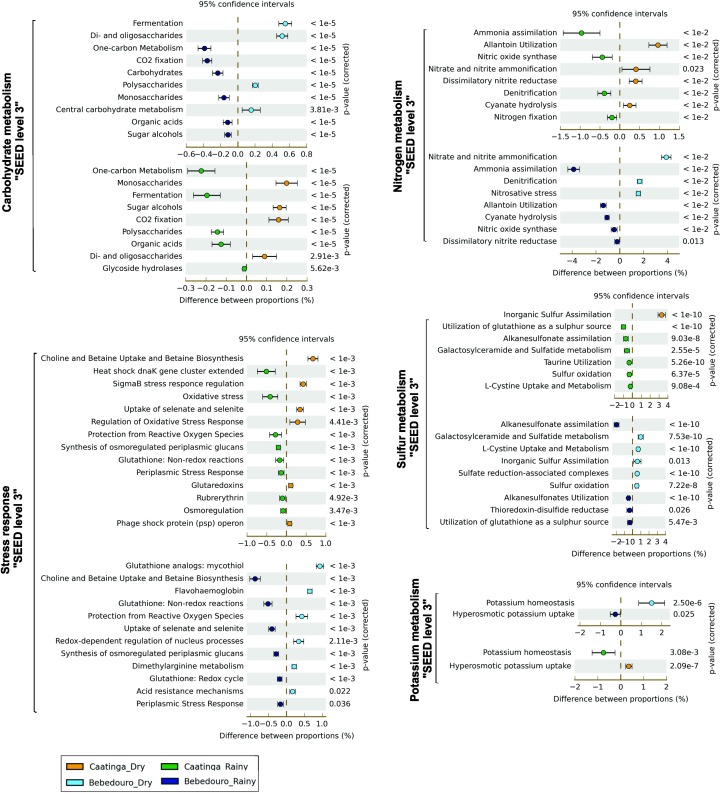
Fisher’s exact test-based comparative analysis of selected metabolic pathways of SEED subsystems (levels 2 and 3) enriched in dry and rainy seasons of CEF and BEF. The Story’s FDR correction was used at *P* < 0.05 filter.

The most striking functional difference between microbial communities from CEF soils of was detected in Virulence, Disease and Defense, followed by Carbohydrates subsystem (SEED level 1, Supplementary Figure S3). The analysis of the functions contained in these subsystems indicated that toxic compounds and antibiotic resistance genes, as well as others likely associated with microbe-microbe competition, were prevalent in moist soils. Interestingly, functions within “Carbohydrates” subsystem overrepresented in dry soils were related with the metabolism of labile C sources, such as monosaccharides (i.e., L-rhamnose, D-ribose, L-arabinose) and oligosaccharides (i.e., maltose/maltodextrin); or osmoprotective sugars (i.e., mannitol and inositol), which also seem to play a role in the drought tolerance (Figure 6 and Supplementary Figure S4; Zahid and Deppenmeier, 2016). On the other hand, moist soils showed an increase of polysaccharide degradation pathways, including xyloglucans (plant polysaccharides) and glycogen. In fact, a vast repertoire of lignocellulolytic enzymes was mined by sequence-based screening from a metagenomic library of a Caatinga soil in the rainy season (Lacerda Júnior et al., 2017). This is expected since the litterfall is the major organic source deposited in the soil of Caatinga forest (Salgado et al., 2015). Also, genes related to “trehalose uptake and utilization” and “trehalose biosynthesis” were more abundant in dry and rainy season soils, respectively (Supplementary Figure S4). Trehalose is an important compatible solute accumulated in bacterial cells as a response to hyperosmotic conditions and as an energy source for long periods of carbon starvation (Argüelles, 2000; Ruhal et al., 2013). The concomitant role of trehalose in drought tolerance and thermoprotection has been established in the soil bacterium *Rhizobium etli* under free-living conditions (Reina-Bueno et al., 2012). It protects and stabilizes membranes and proteins against numerous kinds of stress, including dehydration, oxidation and heat conditions (Crowe, 2007). Thus, this contrasting ability of the microbiome for acquisition and utilization or for biosynthesis of trehalose may be related to the different requirement for osmoadaptation in dry and wet soils.

Additionally, we also explored some of the specific functional categories related to nutrient cycling. Although a subtle statistical difference (*p* < 0.05) was observed in the SEED level 1 “Nitrogen Metabolism” subsystems (Supplementary Figure S3), selective preferences in the biological processes used to uptake and/or convert the available nutrients were showed by lower hierarchical gene annotation. Level 3 of the SEED analysis showed a significant increase in the “nitrogen fixation” and “ammonia assimilation” pathways in moist soils in comparison to a higher abundance of genes involved in “nitrite and nitrate ammonification” (direct uptake of nitrate/nitrite and subsequent reduction to ammonia), and “allantoin utilization” in dry soils (Figure 6, *p* < 0.05). Slight differences were found in the “Sulfur Metabolism” subsystem (Level 1) (Supplementary Figure S3). However, lower level SEED predictions (level 2–3) revealed a higher abundance of genes related to “inorganic sulfur assimilation” in the dry soils in contrast to the enrichment of genes related to “organic sulfur compounds uptake” (especially glutathione as a sulfur source) in the wet season soils (Figure 6). Likewise, potassium (K^+^) metabolism was more abundant in moist soils with the majority of genes involved in K^+^ homeostasis, contrasting with the greatest amount of hyperosmotic K^+^ uptake genes in the dry soils (Figure 6).

The capacity of response to osmotic stress by adaptive mechanisms is an important characteristic that enables drought-tolerant bacteria to survive and proliferate in soils with water shortage (Lebre et al., 2017). As expected, an increase in the functional categories associated with “dormancy/sporulation strategies” and “stress response” was also found in the preserved dry soils (Supplementary Figure S3), with overrepresented sequences involved in the synthesis and uptake systems of compatible solutes including choline and betaine, or encoding putative ion transporters. Comparative analyses using Level 3 “stress response subsystem” (Figure 6) also showed enrichment of several genes encoding bacterial sigma transcription factors responsible for the regulation of bacterial gene expression induced in response to a variety of environmental stresses/physiological signals in the dry soils (Feklístov et al., 2014; Paget, 2015). In contrast, aquaporin Z genes (AqpZ), encoding membrane water channel proteins presumed to play an important role in osmoregulation by accelerating transmembrane water flux in response to abrupt changes in osmotic pressure, were more abundant in rainy season soils. Other general categories, such as “Cell Wall and Capsule,” “membrane transport,” “protein metabolism,” “RNA metabolism,” “Respiration,” “Cofactor, vitamins, prosthetic groups and pigments” categories were more abundant in preserved wet soil of CEF (Supplementary Figure S3).

The enrichment of microbial groups and functional categories related to osmoregulation and nutrient and carbohydrate metabolism shows that the microbial community of preserved soils undergoes an adjustment during the rainy and dry cycles, reflecting differences in the genetic potential for nutrient cycling and carbon acquisition in the environment. This is probably a consequence of seasonal differences in water and nutrient availability since leaf fall in the drought periods is an intrinsic feature of the Caatinga forest; and adaptive processes that ensure long-term resilience over the years of rainfall regime.

### Influence of Agriculture Practices on the Functional Features of Soil Microbiome

As shown, the constant supply of nutrients and water break down the natural water regime in the soils of BEF during the dry season. Hence, the influence of land-use practices outlining the functional traits of the microbial communities was evaluated. A greater relative abundance of “carbohydrates,” “nitrogen metabolism” and “dormancy and sporulation” subsystems (SEED 1) were found in irrigation-affected soils of the dry season. A divergent trend was observed with the enrichment of functions related to water stress response, such as “biosynthesis” and “uptake and utilization” pathways of trehalose, when compared to the differences observed between CEF soils (Figure 6). It has also been shown the prevalence of genes related to di- and oligosaccharides, and polysaccharides degradation (mainly glycogen metabolism).

Interestingly, the overrepresented functions included in the “Stress Response” subsystems in irrigation-affected dry soils were associated with regulation and genetic responses to oxidative stresses such as “mycothiol biosynthesis,” “protection from reactive oxygen species” and “redox-dependent processes” (Figure 6). The increase of these functions suggests a greater functional requirement for cellular protection in response to damage environmental conditions. On the other hand, soils of the rainy season showed a greater enrichment of functions related to osmotic stress, such as “choline and betaine uptake” and “betaine biosynthesis,” and “synthesis of osmoregulated periplasmic glucans” (Figure 6). This unexpected enrichment of functions associated to resistance to osmotic stress in the rainy soils may be the reflection of an adaptation to rewetting-desiccation cycles due to sparse rainfall, in opposition to the continuous water supply by irrigation practices in the soils of the dry season.

Key processes related to the terrestrial N cycle are catalyzed by reductive and oxidative enzymes, in which microbes have a predominant role (Tsoy et al., 2016). Despite the increase of reads assigned to “Nitrogen Metabolism” subsystem (SEED Level 1) in the irrigation-affectedsoils (Supplementary Figure S3), no significant difference (*p* > 0.05) was found in the functional subcategory of “nitrogen fixation” (Figure 6). However, genes encoding enzymes that play roles in protecting from nitrosative stress caused by overproduction of reactive nitric oxide (NO) and NO-derived compounds, nitrate and nitrite ammonification and denitrification processes were overrepresented (Figure 6). The application of N fertilizers to soils has been shown to stimulate nitrogen loss via gaseous emission derived from the denitrification process (Wolsing and Priemé, 2004; Wallenstein et al., 2006; Attard et al., 2011; Yin et al., 2015). The influence of high nutrient input and continuous supply of water coupled with the high abundance of denitrifying bacteria in BEF soils of the dry season, provide highly favorable conditions for denitrification, as shown by Rasche et al. (2011) in temperate forest soils.

The analysis of potassium metabolism at (SEED level-3) revealed a predominance of genes related to K homeostasis in BEF soils of the dry season (Figure 6), probably caused by the high K concentration in such impacted soils (Table 1). Concerning the sulfur metabolism subsystem, organic alkanesulfonate assimilation was the most prevalent route for the environmental sulfur acquisition in the BEF soils of the rainy season. In contrast to a greater abundance of inorganic sulfur assimilation-related genes and other organosulfur reduction and oxidation processes in BEF soils of the dry season (Figure 6). Although soil sulfur content has not been measured, these results indicated differential microbial community strategies to acquire sulfur in the environment in response to impacted conditions.

## Conclusion

Overall, the metagenomics survey showed that natural seasonality and land-use practices differentially affect the composition and functional traits of the soil microbiome from preserved and agriculture-influenced experimental fields of the Caatinga dry forest. Multivariate analysis suggested that environmental factors associated with seasonal cycles (mainly soil moisture) and ferti-irrigation practices (micro- and macronutrients availability) were the major environmental factors driving the microbial community of preserved and agriculture-affected soils, respectively. The strong enrichment of ‘*Candidatus* Koribacter’ and ‘*Candidatus* Solibacter’ raises novel questions about the role of the poorly-known Acidobacteria phylum in fertilization-affected soils and opens the possibility to explore such taxa as bioindicators for agricultural soil management effects in this wide semiarid area. Cellular processes related to osmoregulation and carbon and nutrient cycling are the most impacted functions in preserved soils, indicating different strategies for nutrient cycling and carbon acquisition of the microbiome under different rainfall patterns. However, the nutrient input and disruption of natural water regime by ferti-irrigation practices may impact soil biogeochemical processes linked to microbial processes, such as nitrosative stress and denitrification-related functions, with potential impacts in the soil functioning. This work is an important step toward the understanding of how environment factors affect the soil microbiome in remaining fragments of a seasonal tropical dry forest, occurring exclusively in Brazil. The next step is to understand if these long-term impacts influence the capability of the soil microbial community to maintain functional resilience to perform their normal nutrient cycling and soil structure maintenance processes across the years. These keys ecological drivers may help for the development of sustainable farming systems in dryland ecosystem.

## Author Contributions

GL-J and PF-J carried out the experimental design and soil collection in the experimental fields. GL-J and SdS performed the extraction and quantification of metagenomic DNA. MN performed the bioinformatic analyses of metagenomic data. GL-J, LC, TD, and PF-J performed statistical analyses. The manuscript was written by GL-J (primarily) and PF-J. IM and VO read and refined the final manuscript.

## Conflict of Interest Statement

The authors declare that the research was conducted in the absence of any commercial or financial relationships that could be construed as a potential conflict of interest.
