# Supplementary material for: Land Use and Seasonal Effects on the Soil Microbiome of a Brazilian Dry Forest
Source: Front Microbiol. 2019 Apr 5;10:648. doi: 10.3389/fmicb.2019.00648 (PMC6461016; doi:10.3389/fmicb.2019.00648)
Supplement: Supplementary file 1 [file Data_Sheet_1.docx]

**- SUPPLEMENTARY FIGURES AND TABLES -**

1. **b)**


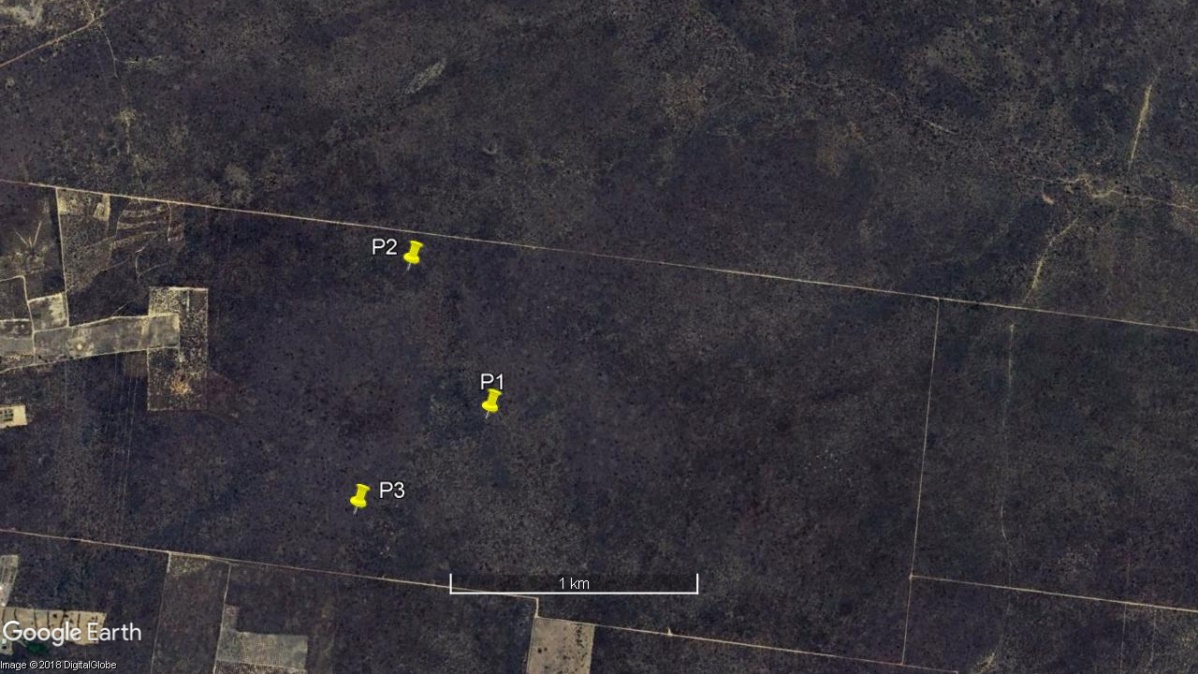

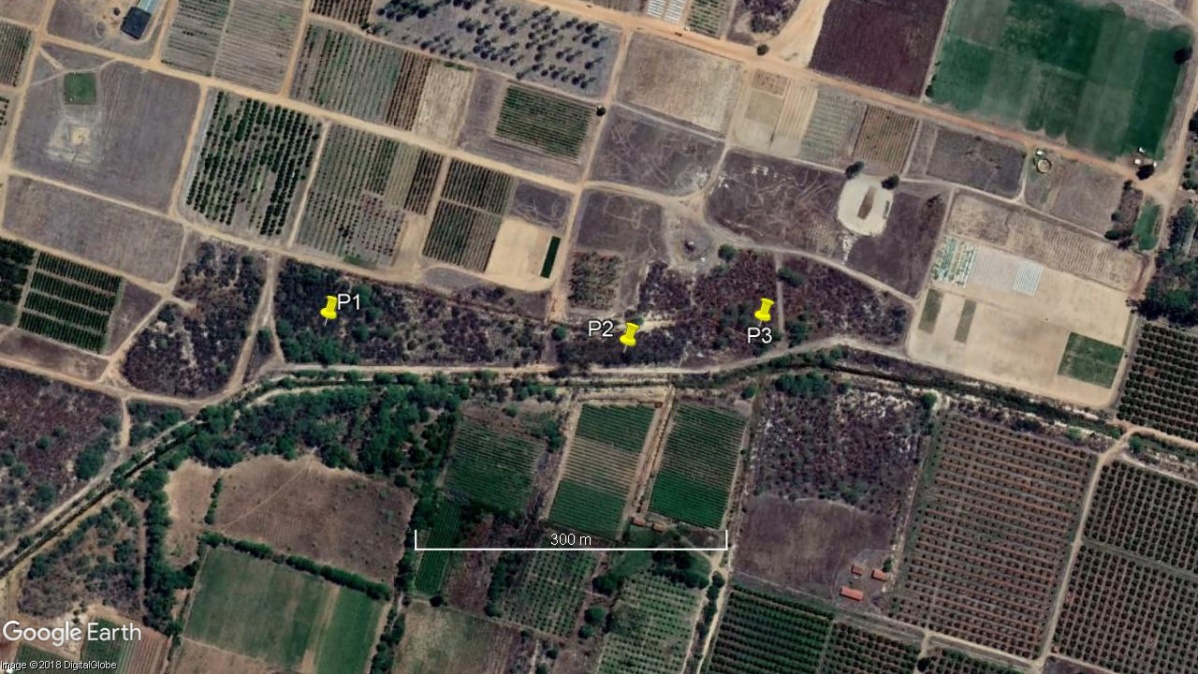


**Supplementary Figure S1.** Satellite image illustrating the sampling sites of the Caatinga **a)** and Bebedouro **b)** experimental stations (geographical coordinates: 09°03'0.94"S/40°19'5.96"W; 09°8'20.28"S/40°18'14.70"W). CEF is a preserved area of the Caatinga dry forest while BEF encompasses Caatinga fragments surrounded by an irrigation perimeter with 6000 hectares used for agricultural crops. Triplicate soil samples (each composed by five subsamples) were randomly collected (depth 0-10 cm) from three different sites (P1, P2 and P3) across CEF and BEF areas during the spring (October 2014) and winter (May 2015), which corresponded to the peak of the dry and wet seasons, respectively. Google earth version 7.3.1 / March 5, 2015.

**
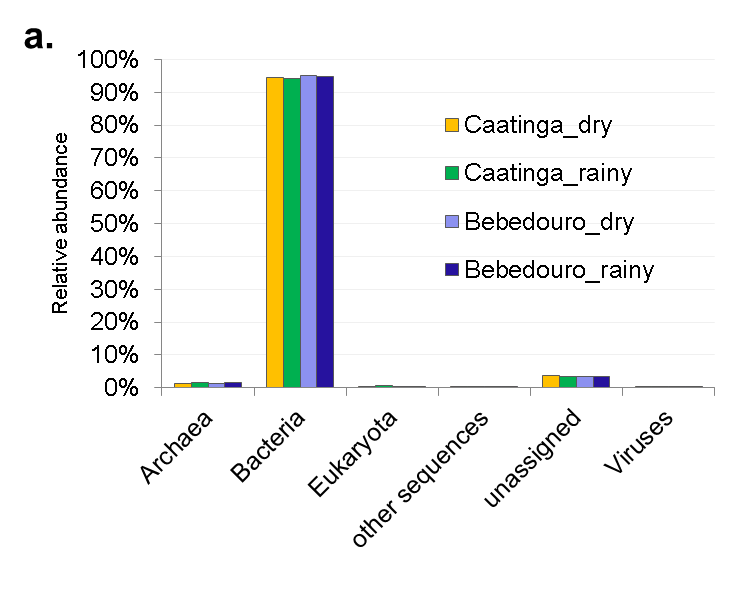
**

**
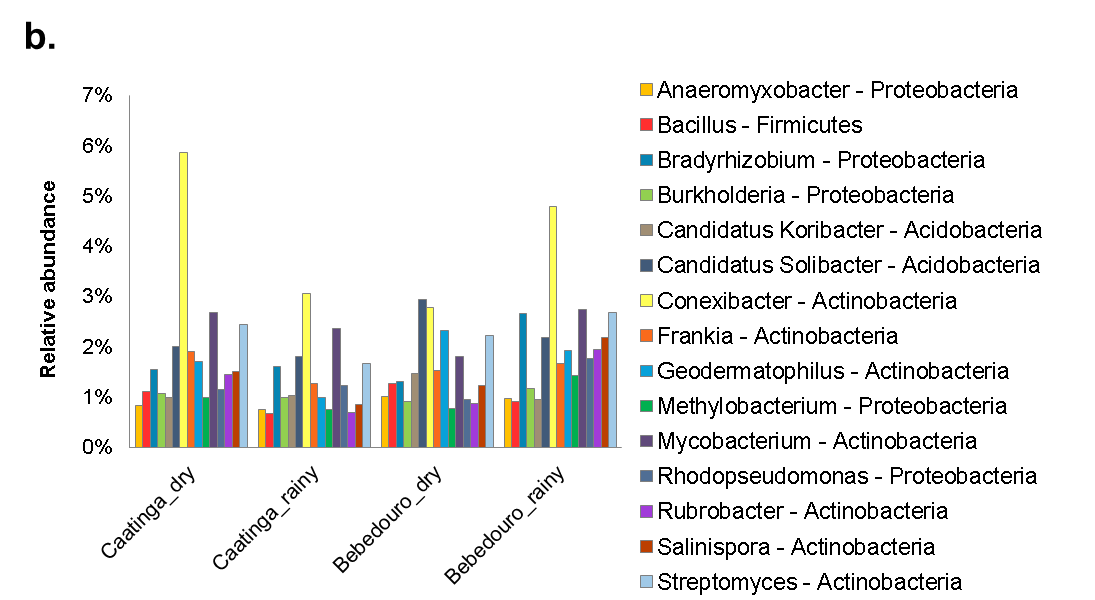
**

**Supplementary Figure S2.** (a) Relative abundance of reads assigned to domains and (b) the 15 most abundant bacterial genera in the metagenomes from preserved CEF and affected BEF soils.

**Supplementary Figure S3.** SEED subsystems (level 1) with significantly different abundances in the dry and rainy seasons of CEF and BEF soil metagenomes. Significance was determined by Fisher’s exact test with Storey’s FDR correction for multiple comparisons; with corrected p < 0.05 filter.

**̽**

**̽**

**̽**

**̽**

**̽**

**̽**

**̽**

**̽**

**̽**

**̽**

**Supplementary Figure S4.** Fisher’s exact test-based comparative analysis of Carbohydrate Metabolism subsystem at level 3 in dry and rainy seasons of CEF and BEF. The Story’s FDR correction was used at P < 0.05 filter.

- **SUPPLEMENTARY TABLE –**

| **Supplementary Table S1**. Characteristics of the EEC and EEB experimental fields from Caatinga dry forest. | | | | | | |
| --- | --- | --- | --- | --- | --- | --- |
| **Biome** | **Coordinates** | **City/State** | **Altitude (m)** | **Soil**  **type** | **Soil temperature** | **Location** |
| Caatinga | 09°03'0.94"S | Petrolina/PE | 375 | Yellow clay | 42°C | Caatinga Experimental Field (CEF) |
|  | 40°19'5.96"W |  |  |  |  |  |
| Caatinga | 09°8'20.28"S | Petrolina/PE | 369 | Yellow clay | 45°C | Bebedouro Experimental Field (BEF) |
|  | 40°18'14.70"W |  |  |  |  |  |

**
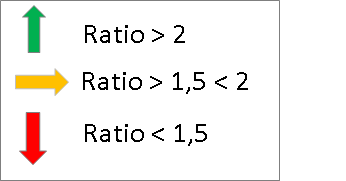
**

| **Supplementary Table S3**. Summary of data processing and annotation by MG-RAST pipeline. | | | | | | | | | | | |
| --- | --- | --- | --- | --- | --- | --- | --- | --- | --- | --- | --- |
| **Metagenome** | **Base pairs (bp)** | **No. of reads pre-QC** | | **No. of reads post QC*** | | **Annotated proteins**** | | **Predicted rRNA genes** | | **GC content (%)** | |
| Bebedouro_dry_P1 | 3,250,432,434 | 31,577,772 | | 29,252,535 | | 6,652,231 (23.23%) | | 7,877 | | 66 ± 9 % | |
| Bebedouro_dry_P2 | 3,739,578,862 | 36,123,405 | | 33,398,215 | | 7,511,887 (22.78%) | | 8,194 | | 66 ± 9 % | |
| Bebedouro_dry_P3 | 3,251,175,922 | 31,704,294 | | 28,983,403 | | 6,127,674 (21.52%) | | 8,625 | | 66 ± 10 % | |
| Bebedouro_rainy_P1 | 4,512,558,394 | 43,524,313 | | 40,100,155 | | 8,747,868 (22.08%) | | 8,584 | | 64 ± 11 % | |
| Bebedouro_rainy_P2 | 4,306,831,471 | 41,489,917 | | 38,285,760 | | 8,968,382 (24.01%) | | 9,402 | | 65 ± 10 % | |
| Bebedouro_rainy_P3 | 2,714,601,800 | 26,718,458 | | 23,469,131 | | 4,960,555 (22.53%) | | 6,654 | | 65 ± 12 % | |
| Caatinga_dry_P1 | 4,020,177,469 | 38,861,913 | | 36,023,156 | | 6,652,231 (23.23%) | | 7,877 | | 65 ± 9 % | |
| Caatinga_dry_P2 | 4,086,971,907 | 39,042,647 | | 36,263,486 | | 6,751,411 (18.91%) | | 8,093 | | 65 ± 10 % | |
| Caatinga_dry_P3 | 3,913,315,921 | 38,066,786 | | 35,028,639 | | 7,480,942 (21.88%) | | 7,294 | | 65 ± 9 % | |
| Caatinga_rainy_P1 | 4,251,639,643 | 41,540,032 | | 38,129,034 | | 7,602,370 (20.46%) | | 8,320 | | 64 ± 10 % | |
| Caatinga_rainy_P2 | 3,658,222,860 | 35,404,431 | | 32,788,829 | | 6,852,175 (23.49%) | | 9,709 | | 64 ± 10 % | |
| Caatinga_rainy_P3 | 3,946,451,848 | 38,065,000 | | 35,251,581 | | 6,933,322 (20.17%) | | 7,025 | | 64 ± 10 % | |
| * Number of remaining reads after quality control by MG-RAST automated processing pipeline. | | | | | | | | |  | |  |
| ** Annotation using SEED database. | | |  | |  | |  | |  | |  |
